# Nanomaterials - Acetylcholinesterase Enzyme Matrices for Organophosphorus Pesticides Electrochemical Sensors: A Review

**DOI:** 10.3390/s90604034

**Published:** 2009-05-26

**Authors:** Arun Prakash Periasamy, Yogeswaran Umasankar, Shen-Ming Chen

**Affiliations:** Department of Chemical Engineering and Biotechnology, National Taipei University of Technology, No.1, Section 3, Chung-Hsiao East Road, Taipei 106, Taiwan; E-Mails: arunprakash.p@gmail.com (A.P); uyogesh@gmail.com (Y.U.)

**Keywords:** acetylcholinesterase, organophosphorus compounds, pesticides, thiocholine, acetylthiocholine, nanomaterials

## Abstract

Acetylcholinesterase (AChE) is an important cholinesterase enzyme present in the synaptic clefts of living organisms. It maintains the levels of the neurotransmitter acetylcholine by catalyzing the hydrolysis reaction of acetylcholine to thiocholine. This catalytic activity of AChE is drastically inhibited by trace amounts of organophosphorus (OP) pesticides present in the environment. As a result, effective monitoring of OP pesticides in the environment is very desirable and has been done successfully in recent years with the use of nanomaterial-based AChE sensors. In such sensors, the enzyme AChE has been immobilized onto nanomaterials like multiwalled carbon nanotubes, gold nanoparticles, zirconia nanoparticles, cadmium sulphide nano particles or quantum dots. These nanomaterial matrices promote significant enhancements of OP pesticide determinations, with the thiocholine oxidation occurring at much lower oxidation potentials. Moreover, nanomaterial-based AChE sensors with rapid response, increased operational and long storage stability are extremely well suited for OP pesticide determination over a wide concentration range. In this review, the unique advantages of using nanomaterials as AChE immobilization matrices are discussed. Further, detection limits, sensitivities and correlation coefficients obtained using various electroanalytical techniques have also been compared with chromatographic techniques.

## 1. Introduction

Acetylcholinesterase (AChE) is an important enzyme present in the synaptic clefts of the central nervous system of living organisms [1]. It hydrolyses the neurotransmitter acetylcholine and facilitates the proper functioning of muscular system [2]. The major role of AChE includes the transmission of the nerve impulses to the cholinergic synapses, involved with memory and Alzhemier's disease. Therefore, AChE has been subject of keen interest for several decades and the detailed studies carried out in the past have revealed that the AChE activity could be significantly inhibited by organophosphorus (OP) pesticides used in agriculture, medicine, industry and chemical warfare agents [3]. The enzyme inhibition mechanism proceeds through the formation of a stable complex through reversible and irreversible reaction of OP pesticides with the active site of AChE [4]. As a result, acetylcholine largely accumulates in the muscular tissues and leads to severe muscular paralysis. Thus the acetylcholine level significantly depends on the availability of active AChE. This ultimately corroborates the need for the detection of OP pesticides in the environment. OP pesticides have been effectively monitored in the past with the aid of various chromatographic techniques [5], which although they provide fruitful results, they are rather time consuming and need very expensive equipment, highly trained personnel and complicated sample pretreatment(s).

In contrast, electrochemical enzyme sensors with high sensitivity, long term stability and low cost detection of specific biological binding events have extensively reduced sampling and testing times in pesticide determinations [6]. Furthermore, such sensors produced precise results under all field conditions. In most AChE sensors, OP detection is mainly based on the irreversible inhibition of AChE activity by OP pesticides [7]. The degree of inhibition has been calculated by comparing the residual activity of the enzyme with the initial activity [4]. Previously, AChE was added directly to solutions to carry out inhibition studies. Though satisfactory results were obtained in such conditions, significant advancements ocurred when AChE was immobilized on modified electrode matrices. However, the enzyme immobilization technique remains rather complicated and often involves very complex matrices [8]. Further, the incorporation of AChE into certain mediator matrices also lowered the stability of the enzyme [9] and reproducibility after pesticide inhibition was also poor in such AChE sensors. In recent years, AChE has been immobilized onto various nanomaterial surfaces in order to improve the response and stability in trace pesticide detection. These nanomaterial matrices include carbon nanotubes (CNTs) [10-15], gold nanoparticles (AuNPs), etc [16-20]. These matrices significantly promote the stability, sensitivity and detection limit in OP pesticide determination in the picomolar (pM) to nanomolar (nM) concentration range. After thorough review of the literature, to our best knowledge, no one has presented a review on application of nanomaterial-based AChE sensors for OP pesticide determination. In this present review, we have summarized the various nanomaterial-based AChE sensors, along with their performance characteristics like sensitivity, linear range and detection limits. In order to demonstrate the significance and the robust performance of such nanomaterials based sensors, we have compared their characteristic features with the complex chromatographic techniques.

## 2. Nanomaterials Based AChE Sensors

In recent years, with high mechanical strength, good conductivity, large surface area and extremely miniaturized size, nanomaterials have been widely employed in electrode modification and electrochemical sensor development. The nanomaterials and their applications have been the subject of many reviews. Pumera *et al.* have reported the major techniques and methods employed in the construction of electrochemical biosensors using nanoscale materials [21]. Gou *et al.* have reviewed the recent advances in the synthesis and electrochemical applications of AuNPs [22]. The most common biosensor-based strategies in both label based metal nanoparticles and label-free (CNT-FET) devices have been reviewed by Kerman *et al.* [23]. Among the various nanomaterials, carbon nanotubes (CNTs) with excellent electrical conductivity, high mechanical strength and good stability have been used extensively in the development of electrochemical biosensors, organic solar cells, transistors and photovoltaic devices [24-28]. Moreover, incorporation of CNTs onto the electrode surface results in the enhanced electron transfer rate with no electrode surface fouling [29]. Recently, Vairavapandian *et al.* have reviewed the various strategies practiced in the preparation and modification of CNTs [30]. They have concluded that incorporation of metal nanoparticles into CNT matrices lead to enhanced catalytic behavior. The readers may refer to similar reviews on CNTs found elsewhere in the literature [31-33]. Other than CNTs, several nanomaterials have been used to modify electrode surfaces. In the following section various nanomaterials, their preparation, the electrode modification methods and the versatile AChE immobilization strategies are presented.

### 2.1. Carbon Nanotubes based AChE Sensors

**Figure 1.**
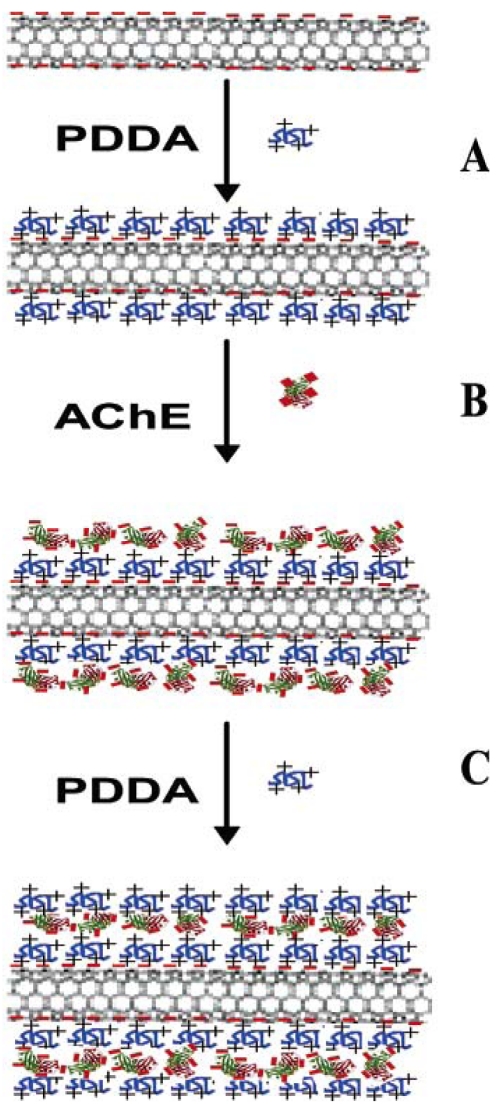
Schematic representation of layer-by-layer electrostatic self-assembly of AChE on MWCNTs: (A) assembling positively charged PDDA on negatively charged MWCNT (B) assembling negatively charged AChE (C) assembling the second PDDA layer (reproduced with permission from Liu *et al.* [12]).

Liu *et al.* earlier reported a highly sensitive flow injection amperometric biosensor for paraoxon detection [12]. The layer-by-layer (LBL) electrostatic self-assembly of AChE on multiwalled carbon nanotubes (MWCNTs) modified GCE was shown in Figure 1. Initially, about 20 μL of the acid treated MWCNTs was cast on the GCE surface and dried. More negative charges were introduced above this MWCNTs modified GCE by dipping in 1 M NaOH solution for 5 min, followed by washing with distilled water twice. This negatively charged MWCNTs/GCE was dipped into an aqueous solution of 1mg·mL^-1^ PDDA containing 0.5 M NaCl for 20 min, which leads to the adsorption of positively charged polycation layer (Figure 1A). Above this polycation layer, a negatively charged AChE layer was adsorbed by dipping the PDDA/MWCNTs/GCE in 0.2 unit·mL^-1^ AChE in Tris-HCl buffer solution pH 8.0 (Figure 1B). Finally, in order to prevent the leakage of AChE from the electrode surface, another PDDA layer was adsorbed above this AChE layer (Figure 1C). This results in a sandwich-like structure of PDDA/AChE/PDDA on the MWCNTs surface. Transmission electron microscopy (TEM) results confirm the formation of layer-by-layer nanostructures on MWCNTs.

Cyclic voltammetry (CV) results show that electrooxidation of thiocholine occurs at much lower oxidation potential (+0.55 V) at MWCNTs modified glassy carbon electrode (GCE), represented as MWCNTs/GCE. Moreover, the electrooxidation current observed at this MWCNTs/GCE was ten times higher than that of bare GCE. The amperometric results further reveal that the response of thiocholine at MWCNTs/GCE was 200 times higher than that of bare GCE. This significant enhancement in the anodic oxidation current of the enzymatic product thiocholine can be attributed to the fast electron transfer and big working surface area of CNTs. They investigated the assay of AChE activity from the plot of current versus acetylthiocholine concentration. The apparent Michaelis-Menten constant (*K*_m_ app) was thus estimated to be 1.75 (mM) using the Lineweaver-Burk plot of 1/I versus 1/[acetylthiocholine]. Where, I represent catalytic current of the analyte. The flow injection analysis (FIA) results show that PDDA/AChE/PDDA/MWCNT/GCE exhibits good reproducibility and stability with no surface fouling effect. The relative inhibition of AChE activity was linear with -log [paraoxon] at the concentration range 1 × 10^-12^ to 0.1 × 10^-9^ M. The detection limit was reported as 0.4 × 10^-12^ M. Their detailed studies show that degree of inhibition was faster and greater with increase in paraoxon concentration and exposure time (optimized as 6 min). The enzyme activity was also regenerated by rinsing with buffer, followed by incubation with pyridine 2-aldoxime methiodide (PAM). They recovered 16.25 and 30 % of enzyme response in a short period of 2 min.

**Figure 2.**
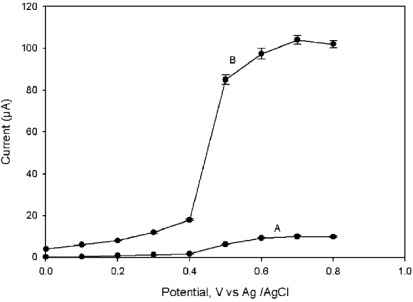
Hydrodynamic voltammogram for 2 × 10^-3^ M thiocholine at (A) unmodified SPE and (B) MWCNTs modified SPE in 50mM phosphate buffer solution (PBS) containing 0.1 M KCl, pH 7.4 (reproduced with permission from Joshi *et al.* [10]).

Similarly, Joshi *et al.* have reported nM paraoxon detection at AChE-functionalized MWCNTs modified screen printed electrode (SPE) [10]. They immobilized AChE by physical adsorption without any mediators. The large surface area and electro-catalytic activity of MWCNTs lowered the over potential for thiocholine oxidation to + 0.2 V. Figure 2 shows the Hydrodynamic voltammograms recorded at unmodified/SPE and MWCNT/SPE for 2 mM thiocholine. It was obvious from the results that significant response occurs at MWCNTs/SPE than at the unmodified SPE.

The linear response of this MWCNT/SPE modified electrode was between 5 × 10^-6^ M - 430 × 10^-6^ M (r^2^ = 0.999) with a sensitivity of 6.018 mA·M^-1^. In contrast, the response of AChE/SPE modified electrode was only 5 %. This result further reveals the contribution of MWCNTs in improving the sensitivity. The *K*_m_ app for AChE was determined to be 0.66 mM. This biosensor also shows good precision and operational stability towards the measurement of acetylthiocholine. The relative inhibition of AChE activity was calculated as a function of paraoxon concentration for 30 min incubation period. The linearity was observed up to 6.9 × 10^-9^ M and the detection limit was 0.5 × 10^-9^ M. Real sample analysis results were also in good agreement (90%), which validates the application of MWCNTs/SPE modified biosensor to a practical problem.

**Figure 3.**
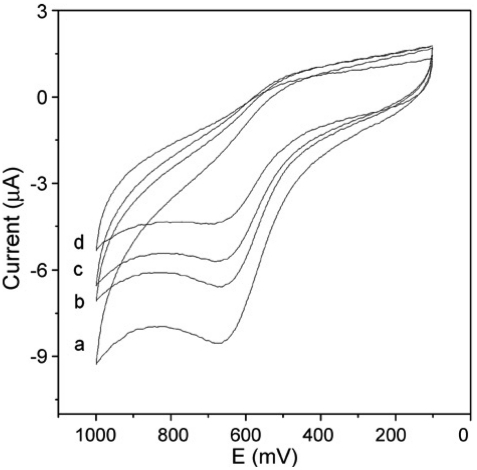
CVs of AChE/MWCNTs-Chi/GCE in pH 7.0 PBS containing 0.4mM acetylthiocholine chloride solution after incubation in (a) 0 × 10^-6^ M (b) 1.5 × 10^-6^ M. (c) 3.5 × 10^-6^ M and (d) 5.2 × 10^-6^ M triazophos solution for 10 min (reproduced with permission from Du *et al.* [14]).

MWCNTs modified electrodes have also been successfully used in triazophos determination. Du *et al.* reported a sensitive, fast and stable amperometric sensor for the quantitative determination of triazophos [14]. They used MWCNTs and chitosan (Chi) as AChE immobilization matrix. They carried out covalent immobilization technique to immobilize AChE at MWCNTs-Chi modified electrodes. The glutaraldehyde was used as a cross linking agent. Their CV results show that the oxidation peak of thiocholine occurs with a maximum height at AChE/MWCNTs-Chi/GCE than AChE/Chi/GCE. This shows that MWCNTs presence lowers the oxidation potential of thiocholine at the MWCNTs-Chi composite electrode. The thiocholine oxidation potential reported in this study (+ 0.2 V) was considerably lower than that reported at MWCNTs/GCE [12]. This AChE/MWCNTs-Chi/GCE have been used for the inhibition of triazophos. The triazophos incubation time was optimized as 10 min, since the interaction between the enzyme and substrate reached saturation after 10 min. Their inhibition studies further reveals that, with increase in triazophos concentration the peak currents decreased at the composite electrode (Figure 3). The inhibition of triazophos was proportional to its concentration in two ranges, from 0.03 × 10^-6^ M to 7.8 × 10^-6^ M and 7.8 × 10^-6^ M to 32 × 10^-6^ M, with correlation coefficients 0.9966 and 0.9960, respectively. The calibration sensitivity and the detection limit are 6.78, 0.87% μM and 0.01 × 10^-6^ M, respectively.

Further, Du *et al.* have reported that incorporation of silica solgel (SiSG) into MWCNTs matrix improved the triazophos detection [15]. The MWCNTs incorporated sol–gel matrix provided a biocompatible microenvironment around the enzyme and efficiently prevents the leakage of the enzyme from the film. CV results show that MWCNTs present in the composite matrix promoted the electron transfer and increased the sensitivity of the sensor towards triazophos detection. Further, with increase in MWCNTs amount, anodic peak current reached a maximum at 20 % (V_MWCNTs_/V). Thermal stability studies show that no loss of enzyme activity occurred in the temperature range 20 – 50 °C. This indicates the excellent activity of the enzyme in this temperature range without any denaturation. Enzyme inhibition studies also show that, up to 12 min the composite electrode displayed a decrease in peak current. This represents the significant AChE inhibition activity by triazophos. However, after 12 min the peak current stabilized. This indicates that binding interactions with active target groups in AChE have reached saturation. However, they found that the maximum value of inhibition of triazophos was not 100%. Since, it was attributable to the binding equilibrium between pesticide and binding sites in the enzyme. Under optimum experimental conditions, the inhibition of triazophos was proportional to its concentration from 0.02 × 10^-6^ M to 1 × 10^-6^ M and from 5 × 10^-6^ M to 30 × 10^-6^ M respectively. The correlation coefficients are 0.9957 and 0.9986. The detection limit was 5.0 × 10^-9^ M. The determination of triazophos in garlic samples showed acceptable accuracy. Fabrication reproducibility of this sensor was also good and stability was also acceptable.

### 2.2. Gold Nanoparticles Based AChE Sensors

As mentioned in Section 2.1, the response, the stability and the enzyme activity are greatly improved on the MWCNTs platform. Apart from CNTs, AuNPs have also been widely used in biosensors for immobilizing biomolecules. With the use of AuNPs, the efficiency and the stability of the pesticide sensor greatly amplified. Moreover, nanoparticle matrix offers much friendly environment to the immobilized enzyme.

**Figure 4.**
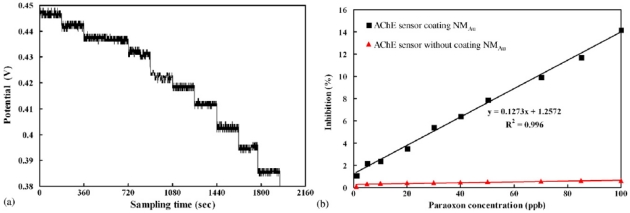
(a) Serial signal response of a LSPR sensor towards paraoxon in the range of 3.63 × 10^-9^ M to 0.36 × 10^-6^ M. The sensor is immersed in 0.05 × 10^-3^ M acetylcholine chloride solution. (b) Calibration plot for the response to paraoxon in the range of 3.63 × 10^-9^ M to 0.36 × 10^-6^ M by two AChE biosensors with/without coating NMAu (reproduced with permission from Lin *et al.* [16]).

Lin *et al.* developed a localized surface plasmon resonance (LSPR) sensor for the determination of paraoxon. They immobilized AChE onto self-assembled gold nanoparticles monolayer (NMAu) [16]. The basic principle of this LSPR sensor is: the light attenuation will be affected as the acetylcholine chloride molecules are captured or reacted by AChE immobilized on the sensor surface. As a result, the decrease of light intensity occurs at the detector. This might be due to the local increase of the refractive index. In the fabrication process, the optimal immobilization conditions for modifying the LSPR sensor are 12.5 mU·mL^-1^ AChE, pH 8.5 and 14 h incubation time, respectively. The serial responding signals from the AChE modified LSPR sensor when immersed in a 0.05 × 10^-3^ M acetylcholine chloride solution for a sequential addition of paraoxon (3.63 × 10^-9^ M to 0.36 × 10^-6^ M) has been shown in Figure 4a. The results show that the responding signals decrease with increasing paraoxon concentration at room temperature. Furthermore, the plot of inhibition (%) versus paraoxon concentration was shown in Figure 4b. This LSPR sensor shows good inhibition towards paraoxon in the concentration range 3.63 × 10^-9^ M to 0.36 × 10^-6^ M. The correlation coefficent was observed to be 0.996 and the detection limit was about 0.85 × 10^-9^ M.

From Figure 4(b), it was obvious that the AChE modified LSPR sensor demonstrates a high response change (14%) with a good linearity in the measurement of paraoxon in the presence of Au (NM) layer. In the absence of NMAu, under the same operating conditions, no significant response change (0.6%) was observed. This shows that the direct binding of AChE on the fiber sensor doesn't occur in the absence of NMAu.

**Figure 5.**
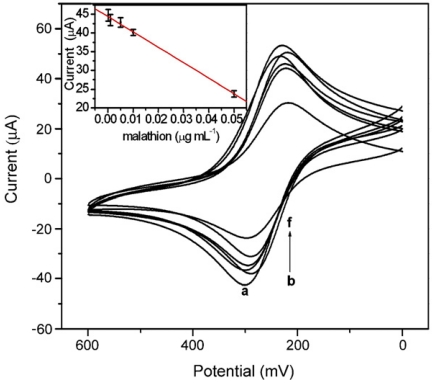
CVs of AChE-Chi/Au in 50 × 10^-3^M [Fe(CN)6]^3/4−^ at 100 mV·s^-1^ after incubation in growth solution containing different malathion concentrations of (a) 0 M (b) 0.30 × 10^-9^ M (c) 3.03 × 10^-9^ M and (d) 30.27 × 10^-9^ M (e) 3.03 × 10^-6^ M and (f) 1.51 × 10^-6^ M. Inset: linear relationship between peak current and malathion concentration (reproduced with permission from Du *et al.* [17]).

Similarly, Du *et al.* developed an AChE electrochemical sensor based on enzyme-induced growth of AuNPs without the addition of any gold nano-seeds [17]. They have successfully used [Fe(CN)_6_]^3−/4−^ as a probe to monitor the electron transfer process. From the CV results they observed that, AuNPs presence increased the peak current and decreased the peak separation. However, the peak current of AChE/Chi/Au modified electrode in growth solution (0.02 % HAuCl_4_ and 3 × 10^-3^ M acetyl thiocholine chloride) increased with incubation time and decreased after 10 min. This may be attributed to the reason that congregated AuNP clusters would have blocked the electron transfer. Similarly, CV and AFM results together confirmed that the nanoparticles size was greatly influenced by acetylthiocholine chloride concentration. With increase in acetylthiocholine chloride concentration, the nanoparticles size increased and leads to aggregation and surface roughness. In contrast, the increase in malathion concentration decreased the AChE activity in the concentration range, 0.30 × 10^-9^ M to 1.51 × 10^-6^ M (R = 0.9989) with a detection limit of 0.06 × 10^-9^ M (Figure 5).

This AChE/AuNPs/Chi sensor showed good precision and reprodubility with a RSD value of 3.3% for five replicate measurements. This clearly demonstrates the applicability of this AuNPs based sensor for pesticide determination.

**Figure 6.**
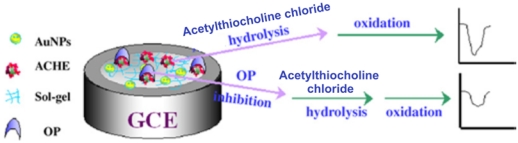
Principle of AChE sensor used for determination of OP compound (reproduced with permission from Du *et al.* [18]).

Du *et al.* proposed a simple method to immobilize acetylcholinesterase (AChE) on silica sol–gel (SiSG)-AuNPs assembly for the sensitive, fast and stable amperometric determination of monocrotophos, an OP pesticide [18]. The principle of the AChE-AuNPs-SiSG sensor used in this study for OP pesticide detection has been shown in Figure 6.

They observed that the large quantities of hydroxyl groups in the sol–gel composite provided a biocompatible microenvironment around the immobilized AChE and thus stabilized its biological activity to a large extent through hydrogen bond formation. A significant change in voltammetric signal occurs at AChE-AuNPs-SiSG/GCE after 10 min incubation in standard solution of monocrotophos. The increase in monocrotophos concentration leads to irreversible inhibition of AChE which was obvious from the considerable decrease in peak current. The inhibition of monocrotophos was proportional to its concentration range from 4.48 × 10^-9^ M to 4.48 × 10^-6^ M and 8.96 × 10^-6^ M to 2.69 × 10^-6^ M. The correlation coefficients are 0.9930 and 0.9985, respectively. The detection limit was 2.69 × 10^-6^ for a 10% inhibition. The inter-assay precision measurements also show that the sensor retained 90% of its initial current response after a 30-day storage period. Similarly, this group developed an AChE biosensor in which AChE was immobilized onto a GCE surface modified with CdTe quantum dots (QDs)/AuNPs and chitosan microspheres (labeled as AChE-CdTe-AuNPs-CM/GCE) [19]. They used this biosensor for the determination of OP pesticide, monocrotophos. The inhibition of monocrotophos was proportional to its concentration in two ranges, from 5.0 × 10^-9^ M to 4.48 × 10^-6^ M and from 9.0 × 10^-9^ M to 0.067 × 10^-6^ M, with a detection limit of 1.34 × 10^-9^ M. Their results reveal that the combination of CdTe QDs and AuNPs promoted electron transfer of AChE and catalyzed the electro-oxidation of thiocholine and thus amplified the detection sensitivity. Furthermore, this nanomaterial based biosensor showed good precision and reproducibility, acceptable stability and accuracy in garlic samples analysis.

**Figure 7.**
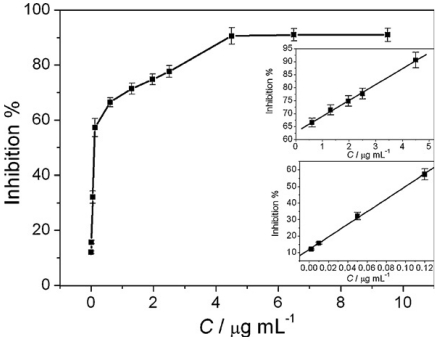
Calibration curve for methyl parathion determination. (Inset) Linear relationships between peak currents and methyl parathion concentrations (reproduced with permission from Gong *et al.* [20]).

Recently Gong *et al.* reported a nanomaterial based AChE biosensor, where they immobilized AChE onto AuNPs–polypyrrole nanowires composite film modified GCE (labeled as AchE-AuNPs-PPy/GCE) [20]. They observed through their studies that the combination of AuNPs and PPyNWs greatly catalyzed the oxidation of the enzymatically generated thiocholine product and increased the detection sensitivity. AChE immobilized at AuNPs and PPyNWs nanocomposite platform was greatly inhibited by methyl parathion in the concentration range of 0.019 × 10^-6^ M to 0.45 × 10^-6^ M and 1.90 × 10^-6^ M to 17.10 × 10^-6^ M (Figure 7). The detection limit was 7.60 × 10^-6^ M.

The selectivity of AchE-Au-PPy/GCE has been investigated through interference studies. No obvious inhibition was observed at this AchE-Au-PPy/GCE towards the electroactive nitrophenyl derivatives such as nitrobenzene, nitrophenol and other oxygen-containing inorganic ions (PO_4_^3-^, SO_4_^2-^, NO_3_^−^). However, only slight variation in the acetylthiocholine peak currents was observed.

### 2.3. Zirconia Nanoparticles Based AChE immunosensors

**Figure 8.**
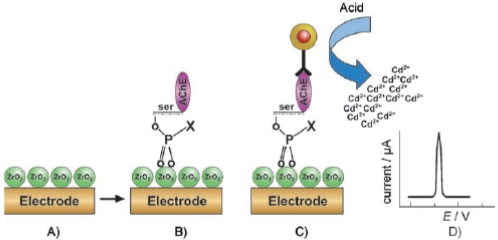
The principle of electrochemical immunosensing of phosphorylated AChE, (A) ZrO_2_ NPs modified SPE; (B) Selective capturing phosphorylated AChE adducts; (C) Immunoreaction between bound phosphorylated AChE adducts and QD-labeled anti-AChE antibody; (D) dissolution of nanoparticles with acid following an electrochemical stripping analysis. (Reproduced with permission from Liu *et al.* [34]).

Liu *et al* developed a ZrO_2_ NPs-based electrochemical immunosensor for the detection of phosphorylated AChE, a potential biomarker to OP pesticides and chemical warfare nerve agents [34]. The principle of the electrochemical immunosensing of phosphorylated AChE adducts based on a QD label and a ZrO_2_ NPs-modified SPE was shown in Figure 8.

In this study, ZrO_2_ NPs were used as selective sorbents to capture the phosphorylated AChE adduct. The quantum dots (ZnS@CdS, QDs) were used as tags to label monoclonal anti-AChE antibody for the measurement of immunorecognition events. Initially, ZrO_2_ NPs were electrochemically deposited onto bare SPE. Then this bare SPE was transferred to aqueous electrolyte containing ZrOCl_2_ (5.0 × 10^-3^ M) and KCl (0.1M) and 10 consecutive cycles were performed in the potential between –1.1 and +0.7 V (versus Ag/AgCl) at a scan rate of 20 mV·s^-1^. The SPE modified with ZrO_2_ NPs, (ZrO_2_/SPE) was thus obtained. The phosphorylated AChE and QDs were captured at ZrO_2_/SPE. Electrochemical stripping analysis of the metallic component (cadmium) followed by an acid-dissolution step was carried out to determine the captured QD tags on the SPE. In this study, Paraoxon was used to prepare the phosphorylated AChE adducts. The phosphorylated AChE adduct was characterized by Fourier transform infrared spectroscopy (FTIR) and mass spectroscopy. The voltammetric response of the immunosensor is highly linear over the range of 10 × 10^-12^ M to 4 × 10^-9^ M phosphorylated AChE and the limit of detection was estimated to be 8.0 × 10^-12^ M, respectively. Though this ZrO_2_ nanoparticle modified immunosensor is extremely suitable for the successful monitoring of OP pesticides, good selectivity can be achieved further through the implementation of QD-tagged anti-phosphorylated AChE antibody.

### 2.4. CdS Nanoparticles based Photoelectrochemical AChE Sensors

Pardo-Yissar *et al.* carried out a versatile photoelectrochemical approach for the detection of OP pesticides [35]. They have successfully developed AChE/CdS NPs hybrid system for the photoelectrochemical detection of AChE inhibitors. CdS NPs of 3 nm diameter were capped with a protecting monolayer of cysteamine and mercaptoethane sulfonic acid. The X-ray photoelectron spectroscopy (XPS) analysis reveals that the 84% of the Cd^2+^ surface groups were linked to the thiolated molecules and the ratio between the cysteamine and thiol sulfonate units were about 1:10, respectively. From their further studies, it was obvious that the capped CdS NPs are covalently linked to a gold electrode functionalized with *N*-hydroxysuccinimide active ester cysteic acid. Quartz crystal microbalance (QCM) measurements were carried out to investigate the analogous association of the CdS NPs on a gold quartz crystal. The results indicate that the binding of the CdS NPs to the gold surface involves a change of Δf = 140 Hz that corresponds to a surface coverage of 5.7 × 10^12^ particles·cm^-2^. On this CdS NPs linked gold substrates, AChE was covalently linked using glutaraldehyde. Parallel microgravimetric QCM measurements demonstrate that nanoparticles are associated with each AChE unit. It was obvious from the surface coverage value obtained for AChE (3.9 × 10^-12^ mol·cm^-2^).

**Figure 9.**
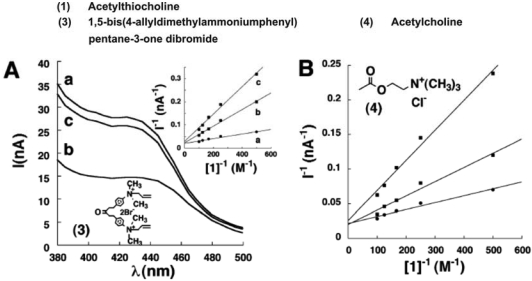
(A) Photocurrent spectra corresponding to the CdS/AChE system in the presence of acetylthiocholine, 10 × 10^-3^M (a) without the inhibitor; (b) upon addition of 10 × 10^-6^ M inhibitor; (c) after rinsing the system and excluding of the inhibitor. Inset shows the Lineweaver-Burke plots corresponding to the photocurrent at variable concentrations of acetylthiocholine, in the presence of inhibitor. (a) 0 × 10^-6^ M, (b) 10 × 10^-6^ M, (c) 20 × 10^-6^ M. Data were recorded in 0.1 M PBS, pH = 8.1, under argon atmosphere. (B) Lineweaver-Burke plots corresponding to the photocurrent at variable concentrations of acetylthiocholine, in the presence of (a) 0 × 10^-3^ M, (b) 1 × 10^-3^ M, (c) 2 × 10^-3^ M of acetylcholine. Data were recorded in 0.1 M phosphate buffer, pH = 8.1, under argon atmosphere (reproduced with permission from Pardo-Yissar *et al.* [35]).

Figure 9A shows the photocurrent spectra recorded at CdS/AChE for different concentrations of acetylthiocholine. The results indicate that the photocurrent originates from the excitation of the CdS NPs and it occurs only in the presence of acetylthiocholine. On the other hand no photocurrent was noticed when the CdS NPs monolayer that lacks AChE was irradiated in the presence of acetylthiocholine. Moreover, the irradiation of an AChE directly linked electrode in the presence of acetylthiocholine also does not yield any photocurrent. Thus they confirmed that the photocurrent generation was due to the AChE-catalyzed hydrolysis of acetylthiocholine. Furthermore, the reason for the steady state photocurrent might be due to the oxidation of thiocholine by holes, which eliminates the electron-hole recombination. Their detailed studies confirm that the generated photocurrent remains stable for 1 h. The photocurrent spectra were recorded at AChE-functionalized CdS NPs for addition of acetylthiocholine and 10 × 10^-3^ M 1,5-bis(4-allyldimethylammoniumphenyl)pentane-3-one dibromide (Figure 9A). The spectra results show that, increase in inhibitor concentration decreases the photocurrent. However, the photocurrent restored when the inhibitor was washed off from the cell after acetylthiocholine addition. The photocurrent decrease observed in the presence of the inhibitor has been attributed to the lower yields for the biocatalyzed formation of thiocholine and less efficient removal of the valence-band holes. In the presence of acetylthiocholine, AChE linked CdS NPs possess a *K*_m_ app value of 5 × 10^-3^ M. Thus this study clearly demonstrates that enzyme inhibitors decrease the photocurrents and thus the CdS NPs/AChE system acts as a biosensor for the respective inhibitor.

### 2.5. CdS Quantum Dots based AChE Sensors

Cadmium sulphide Quantum dots (QCdS) have also been effectively employed in sensing OP pesticides like trichlorfon. Poly (*N*-vinyl-2-pyrrolidone) (PVP)-capped CdS quantum dots (QCdS-PVP) have been synthesized by Li *et al.* using CdCl_2_ and Na_2_S in the presence of PVP [36]. AChE was immobilized onto this QCdS-PVP matrix incorporated GCE surface. The resulting AChE/QCdS-PVP/GCE sensor was used for the detection of OP pesticide, trichlorfon. TEM results show that, the QCdS-PVP particles are homogeneously distributed and they possess an average size of 2 – 4 nm.

**Figure 10.**
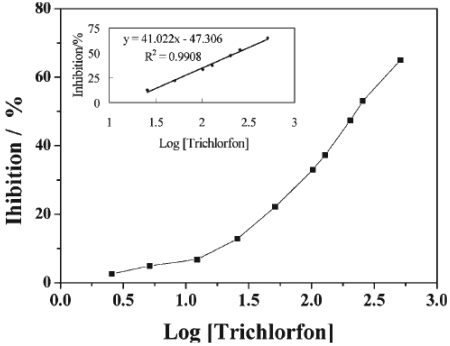
Inhibition plot of the AChE/ QCdS-PVP /GCE biosensor by trichlorfon after 5 min incubation. Measurement conditions: PBS 0.1 M, pH 7.0; E = +0.6 mV (vs. SCE); 2.0 × 10^-5^ M acetylthiocholine iodide (reproduced with permission from Li *et al.* [36]).

Amperometric studies reveal that the AChE/QCdS-PVP/GCE sensor showed a good response to the added acetylthiocholine chloride in the linear range 2.0 × 10^-5^ M to 7.0 × 10^-4^ M. The linear regression equation was I = 0.0724 + 0.0917, respectively. The correlation coefficient was 0.9983 and detection limit was 5 × 10^-6^ M for S/N=3. From the inhibition plot in Figure 10, it was understandable that the relative inhibition of AChE activity increased with the concentration of trichlorfon in the range 1 × 10^-8^ to 2 × 10^-6^ M and is linear with log [trichlorfon] in the concentration range from 1 × 10^-7^ M to 2 × 10^-6^ M. The detection limit was 4.8 × 10^-8^ M. This AChE/QCdS-PVP/GCE sensor showed good reproducibility and it can be regenerated simply by buffer dipping for a limited inhibition. This successful application of QD based pesticide sensors towards OP compounds detection might encourage the researchers to implement other form of nanomaterials including nano cones, nano rods, nano wires, core shell nanoparticles, etc.

## 3. Advantages of Using Nanomaterials for AChE Immobilization

With their wide range of advantageous properties, nanomaterials have been extensively used as potent immobilization matrices for AChE. The various nanomaterials matrices used in AChE biosensors have been discussed in Section 2. The linear range, the detection limit and the correlation coefficient values obtained using various electroanalytical techniques have been presented in Table 1. It is notable that wholly on a comparative basis; these electroanalytical values have been compared with various chromatographic techniques (which are not sensors) and organic phase enzyme electrodes (OPEE) based biosensors (without nanomaterials and AChE).

From Table 1, it was obvious that compared to versatile chromatographic techniques, nanomaterial based AChE sensors are more suitable for OP pesticides determination in wide linear range with the detection limits in nM to pM range. Among various nanomaterial based AChE sensors, the lowest detection of paraoxon (0.4 × 10^-12^ M) has been reported at PDDA/AChE/PDDA/MWCNT/GCE (See Table 1). Similarly, nM detection of paraoxon (0.5 × 10^-9^ M) has also been reported at MWCNTs modified SPE and OPEEs discussed in Table 1. However, by using chromatographic techniques LC-APCI-MS and HPLC, the detection limit of paraoxon has been observed only to be 0.08 × 10^-6^ M and 0.50 × 10^-6^ M respectively. Thus compared to LC-APCI-MS and HPLC, CNTs modified electrochemical sensors have greatly facilitated the detection of paraoxon in pM and nM range. Other than paraoxon, trace amounts of triazophos has also been determined at nanomaterial modified electrodes, AChE/MWCNTs-Chi/GCE and MWCNTs/SiSG/GCE. The detection limits were about 0.01 × 10^-6^ and 5.0 × 10^-9^ respectively. Nevertheless by LC-APCI-MS technique the lowest detection limit of triazophos reported is only about 0.02 × 10^-6^ M. This shows that nM detection of triazophos could be achieved predominantly at CNTs modified electrochemical sensors rather than LC-APCI-MS technique. Moreover, excluding CNTs various other nanomaterial matrices have also been effectively employed for the determination of trace amounts of OP pesticides. For example, ZrO_2_ NPs modified SPE has been employed for the detection of phosphorylated AChE adducts in pM range (See Table 1). AuNPs modified electrodes have also been used for trace OP pesticides detection (See Table 1). For instance, AChE/AuNPs/Chi modified electrode has been used to detect 0.06 × 10^-9^ M malathion. This detection limit has been found to be much lower than that measured using chromatographic techniques MEKC, GC, MSPD combined GC and OPEEs discussed in Table 1. Though AuNP-modified matrices displayed significant improvement in OP pesticide determination, there is always possibility for thiocholine, the AChE mediated hydrolysis product, to bind with the AuNP-modified surface (due to the greater affinity of thiol (-SH) groups towards metallic gold), which might reduce the performance of the sensor [45]. Finally, the AChE/QCdS-PVP/GCE has also been used to monitor the OP pesticide, trichlorfon in nM range. This shows that QDs can also be employed for trace OP pesticide determination. This overall shows that majority of the nanomaterial matrices are extremely suitable for AChE immobilization and for trace OP pesticide determinations.

On the other hand, aside from nanomaterial-based AChE sensors, many electrochemical sensors (without nanomaterials) [46], magnetoeleastic sensors [47], OPPE based biosensors [48-49] and immunosensors [50,51] have also been successfully used in the past years for trace pesticide determinations. The performance characteristics of these devices have also been excellent and promising. In particular, OPEEs have been widely used for the detection of OP pesticides which dissolve only in non-aqueous solvents. The non aqueous solvents generally used include acetonotrile, chloroform, dioxane and hexane. Abundant work has been done in this field using various monoenzymatic and bienzymatic systems [52,53]. For instance, the performance characteristics obtained at the monoenzymatic and bienzymatic systems reported by Campanella *et al.* have been presented in Table 1 [44]. They have used kappa-carrageenan gel to immobilize enzymes like tyrosinase, butyrylcholinesterase and choline oxidase. This kappa-carrageenan gel remains as a promising matrix for immobilizing enzymes and thus helps to detect the OP pesticides like paraoxon, malathion and parathion-ethyl in the μM range. The detection limits were in nM range (See Table 1).

### 3.1. Comparison of Real Sample Analysis of OP Pesticides at Nanomaterial Modified Electrodes and the Chromatographic Techniques

The real time analysis in predominant field conditions is much needed for the robust performance of a nanomaterial-based electrochemical sensor. Nevertheless, only a few nanomaterial based electrochemical sensors have reported real sample analysis. We have compared the real sample analysis data obtained from those nanomaterial based electrochemical sensors with the chromatographic techniques. Among various nanomaterial modified electrodes, MWCNTs/SPE has been employed for the analysis of paraoxon in lake water samples [10]. Good recovery of more than 90% has been achieved using MWCNTs/SPE. Whereas, by using LC-APCI-MS the recovery of paraoxon in three honey bee samples were only about 76%, 80 % and 82 % respectively. Thus compared to LC-APCI-MS, CNTs modified SPE helps for the excellent recovery of paraoxon in real samples. However, we cannot rule out the good recovery obtained for paraoxon using HPLC reported by Corcia *et al.* They have successfully used 250 mg graphitized carbon black, also called a Carbopack B catridge, to isolate the paraoxon present in three drinking water samples with different volume (1 L, 2 L and 4 L) [43]. The paraoxon recoveries in the above mentioned drinking water samples were found to be about 100 %, 97 % and 99 %, respectively. The use of Carbopack B catridge also has excellent advantages like good recovery and stepwise elution eliminating the interferences with the analysis of acidic compounds by nonacidic compounds. However, the complete analysis of paraoxon in bulk water samples using this technique rather relies on hazardous and more expensive solvents as eluant systems. Other than paraoxon determination, MWCNTs modified electrodes have also been employed for the determination of triazophos in real samples. The MWCNTs/SiSG/GCE has been employed to detect triazophos in garlic samples [15]. The results obtained at this CNTs modified electrode have been compared with that obtained using HPLC. The relative deviations between the two methods were only in the range of 8.0 % to -8.6 %. The results are in good acceptable agreement. Thus the MWCNTs/SiSG/GCE could be predominantly employed to the determination of triazophos in real samples, since the recovery and analysis time is more rapid in the case of this CNTs modified electrode. However, using chromatographic techniques better recovery is achieved only after a substantial period of time. For instance, using GC technique 95.5 % recovery of triazophos present in pasteurized milk samples have been achieved only after 14 days [37]. Besides, triazophos determination in real samples, nanomaterial modified electrodes have also been effectively employed in the determination of OP pesticide, monocrotophos. Du et al have carried out recovery tests using AChE-CdTe-AuNPs-CM/GCE in garlic samples for the determination of monocrotophos [19]. They added different amounts of monocrotophos into garlic samples of 0.1M PBS. They obtained good recoveries from 95.0% to 102.3%. The average precision is ±6.0%. Furthermore, good recovery of phosphorylated AChE in human plasma samples have been achieved using ZrO_2_ NPs/SPE based immunosensor [34]. The spiked samples show a promising recovery of about 106 % and 109 %. Thus with good recovery and rapid analysis procedures, the above discussed nanomaterial modified electrodes remains more promising for the real sample analysis and thus validate their real time applications in predominant field conditions.

## 4. Conclusions

In recent years, with the excessive use of unwanted amounts of pesticides the risks of human health care and the environment have risen to critical levels. In particular, OP pesticides have been widely employed in agriculture to control many pests. Though OP compounds have their unique advantages of easy degradation in the environment, their excessive usage could cause drastic effects like respiratory, myocardial and neuromuscular transmission impairment [54]. Thus the low level detection of OP pesticides in the environment as well in the real samples is much required. On the other hand, AChE based sensors have been largely used towards trace OP pesticide detection because of their fast responses accompanied by inherent ability to bind for specific target molecules. Further, they provide real-time qualitative and quantitative information about the composition of a sample with minimum sample preparation [55]. Despite of extensive progresses made in electrochemical sensor research, not many AChE sensors find trace OP pesticide detection application in real samples. The advances in nanotechnology together with the beneficial properties of nanomaterials have opened new horizons for the pesticide sensors development. With the implementation of nanomaterials including CNTs, AuNPs, ZrO_2_ NPs, CdS NPs and QCdS as immobilization matrices, dramatic enhancement in the electrocatalytic activity with very high sensitivity towards OP pesticide detection is realized. Moreover, most of the AChE incorporated nanomaterial matrices showed excellent response on storage. In addition, nM-pM amounts of OP pesticides detection have been reported at several nanomaterials modified electrode surfaces. This robustly illustrates the inherent ability of the nanomaterial matrices to promote OP pesticide detection. The long storage stability of the nanomaterial modified sensors draws additional interest among the researchers and this might provoke more workings in this related field in the near future.

## Figures and Tables

**Table 1.** Comparison of performance characteristics of nanomaterial based AChE sensors with chromatographic techniques and OPEEs.

**Immobilization method**	**Electrode type**	**Techniques [Incubation time]**	**OP (Linear conc. in M)**	**Detection limit in M [correlation coefficient]**	**Ref.**
Physical adsorption	MWCNTs/SPE	Amperometry [30 min]	Paraoxon (1.0 × 10^-9^ to 6.9 × 10^-9^)	0.5 × 10^-9^ [0.9859]	[10]
LBL self assembling technique	PDDA/AChE/PDDA/MWCNT/GCE	FIA [6 min]	Paraoxon (1 × 10^-12^ to 0.1 × 10^-9^)	0.4 × 10^-12^	[12]
Covalent immobilization using glutaraldehyde as cross linking agent	AChE/MWCNTs-Chi/GCE	CV [10 min]	Triazophos (0.03 × 10^-6^ to 7.8 × 10^-6^ and 7.8 × 10^-6^ to 32 × 10^-6^)	0.01 × 10^-6^ [0.9966, 0.9960]	[14]
Physical entrapment	MWCNTs/SiSG/GCE	CV [12 min]	Triazophos (0.02 × 10^-6^ to 1 × 10^-6^ and 5 × 10^-6^ to 30 × 10^-6^)	5.0 × 10^-9^ [0.9957 and 0.9986]	[15]
Self assembled monolayer (SAM)	Optical fibers modified with self assembled AuNPs	LSPR [14 h]	Paraoxon (3.63 × 10^-9^ to 0.36 × 10^-6^)	0.85 × 10^-9^ [0.996]	[16]
Physical adsorption	AChE/AuNPs/Chi	CV [10 min]	Malathion (0.30 × 10^-9^ to 1.51 × 10^-6^)	0.06 × 10^-9^[0.9989]	[17]
Physical entrapment	AChE-AuNPs-SiSG	CV [10 min]	Monocrotophos (4.48 × 10^-9^ to 4.48 × 10^-6^ and 8.96 × 10^-6^ to 2.69 × 10^-6^)	2.69 × 10^-6^ [0.9930 and 0.9985]	[18]
Covalent immobilization	AChE-CdTe-AuNPs-CM/GCE	CV[8 min]	Monocrotophos (5.0 × 10^-9^ to 4.48 × 10^-6^ and from 9.0 × 10^-9^ to 0.067 × 10^-6^)	1.34 × 10^-9^ [0.9927 and 0.9945]	[19]
Physical adsorption	AChE–AuNPs–PPy/GCE	CV [12 min]	Methyl parathion (0.019 × 10^-6^ to 0.45 × 10^-6^ and 1.90 × 10^-6^ to 17.10 × 10^-6^)	7.60 × 10^-6^ [0.9992 and 0.9989]	[20]
Affinity immobilization	ZrO_2_ NPs/SPE	Striping voltammetry	Phosphorylated AChE adducts (10 × 10^-12^ - 4 × 10^-9^)	8.0 × 10^-12^ [0.9955]	[34]
Covalent immobilization using glutaraldehyde	AChE/CdS NPs	Photocurrent spectra	1,5-bis(4- allyldimethylammoniumphenyl) pentane-3-one dibromide	-	[35]
Physical entrapment	AChE/ QCdS-PVP /GCE	Amperometry [5 min]	Trichlorfon (0.1 × 10^-9^ - 2 × 10^-6^)	0.5 × 10^-9^ [0.9908]	[36]
-	-	Gas chromatography (GC)	Methyl parathion Malathion Triazophos	0.04 × 10^-6^ 0.03 × 10^-6^ 0.08 × 10^-6^	[37]
-	-	Automated on-line solid-phase extraction (OSP-2) and thermospray mass spectrometry (LC-TSP- MS)	Malathion (0.076 × 10^-6^ to 6.05 × 10^-6^ M)	0.09 × 10^-6^ [0.9831]	[38]
-	-	GC	Malathion	3.02 × 10^-9^ [0.997]	[39]
-	-	Liquid chromatography -atmospheric pressure chemical ionization-mass spectrometry (LC-APCI-MS)	Malathion Paraoxon Triazophos	0.02 × 10^-6^ 0.08 × 10^-6^ 0. 02 × 10^-6^	[40]
-	-	Automated solid phase extraction and micellar electrokinetic capillary chromatography (MEKC)	Malathion	0.15 × 10^-6^ [0.9987]	[41]
-	-	Matrix Solid-Phase Dispersion (MSPD) and GC	Parathion methyl Malathion	4.0 × 10^-9^ 9.0 × 10^-9^	[42]
-	-	High pressure Liquid chromatography (HPLC)	Paraoxon	0.50 × 10^-6^	[43]
-	Tyrosinase enzyme immobilized in kappa-carrageenan gel	Inhibition [15 min]	Paraoxon 0.01 × 10^-6^ to 0.1 × 10^-6^ Malathion 0.01 × 10^-6^ to 0.1 × 10^-6^ Parathion-ethyl 0.01 × 10^-6^ to 0.1 × 10^-6^	5.0 × 10^-9^ 5.0 × 10^-9^ 5.0 × 10^-9^	[44]
-	Butyrylcholinesterase and choline oxidase immobilized in kappa-carrageenan gel	Inhibition [15 min]	Paraoxon 0.03 × 10^-6^ - 0.5 × 0^-6^ Malathion 0.03 × 10^-6^ to 0.025 ×10^-6^ Parathion-ethyl 0.02 × 10^-6^ to 0.025 ×10^-6^	15.0 × 10^-9^ 15.0 × 10^-9^ 2.0 ×10^-9^	[44]
